# Supplementary material for: Should I study or should I go (to sleep)? The influence of test schedule on the sleep behavior of undergraduates and its association with performance
Source: PLoS One. 2021 Mar 10;16(3):e0247104. doi: 10.1371/journal.pone.0247104 (PMC7946303; doi:10.1371/journal.pone.0247104)
Supplement: Attachment — Submitted filename: MS.v2-Responses.docx [file pone.0247104.s001.docx]

**Academic Editor**

Please ensure that your manuscript meets PLOS ONE's style requirements, including those for file naming.

The manuscript has been revised according to the journal style requirements.

Please consider changing the title so as to meet our title format requirement

We extended the title with a description of the aims of the study.

Please clarify (on line 228) whether you can conclude about students' lack of sleep or their reported lack of sleep.

Thank you for the observation. We modified the expression to better describe our findings (line 241).

Please provide additional details regarding participant consent. In the ethics statement in the Methods and online submission information, please ensure that you have specified whether consent was informed.

The use of informed consent was included in line 90.

We note that you have stated that you will provide repository information for your data at acceptance. Should your manuscript be accepted for publication, we will hold it until you provide the relevant accession numbers or DOIs necessary to access your data.

The information was included in the Cover Letter.

**Reviewer #1**

My main concern is that the size of the effect is small; in survey 1, 1 hour more sleep meant 5% increase in correct answers and in survey 1, 1 hour more sleep was only associated with 1.4% increase in correct answers. These numbers may not be significant to change the overall grade for the subject. Do the authors have data instead on whether more students failed if got less sleep? Or if the overall grade (ie, A to B, B to C) was effected because of less sleep?

Thank you for this observation. We did again the analyses using logistic regressions to explain the correct answers rate. The results did not change, but these new analyses explain a greater proportion of the deviance, better modeling the association between sleep and performance (lines 124-131 and 168-177 in Results, table 1, and lines 208-211 in Discussion). The abstract was modified accordingly (lines 32-34).

In Uruguay, grades are given in figures on a scale from 0 to 12.The fair lowest passing grade is 3 and corresponds to 60% achievement, while 91% achievement corresponds to grade 10. This complexity of the Uruguayan grading system prevented us from including the grades. Instead, we estimated the correct answer rate for extreme sleep durations of 0h and 8h (lines 130-131 and 176-177). The smaller change in survey #2 is now discussed in lines 213-216.

Lines 185 and 186: not sure that 'short sleep ratio' is previously defined; would find different wording.

The expression was replaced (lines 194-195).
