# Supplementary material for: Should I study or should I go (to sleep)? The influence of test schedule on the sleep behavior of undergraduates and its association with performance
Source: PLoS One. 2021 Mar 10;16(3):e0247104. doi: 10.1371/journal.pone.0247104 (PMC7946303; doi:10.1371/journal.pone.0247104)
Supplement: Attachment — Submitted filename: MS.v3-Response to Reviewers.docx [file pone.0247104.s002.docx]

**Reviewer #1**

The authors have better explained the grading system in Uruguay and why they cannot provide 'grades' per se. This explanation would also be helpful to other readers and therefore my final recommendation is that the sentences written to me about grading be added to the Discussion. I think important as clearly the predicted grades on line 131 represent 'failure' v. 'passing.'

As suggested, sentences describing the grading system in Uruguay were included in the discussion (lines 211-216).
